# A Dewetted‐Dealloyed Nanoporous Pt Co‐Catalyst Formed on TiO_2_ Nanotube Arrays Leads to Strongly Enhanced Photocatalytic H_2_ Production

**DOI:** 10.1002/asia.201901545

**Published:** 2020-01-07

**Authors:** Lei Ji, Davide Spanu, Nikita Denisov, Sandro Recchia, Patrik Schmuki, Marco Altomare

**Affiliations:** ^1^ Department of Materials Science WW4-LKO University of Erlangen-Nuremberg Martensstrasse 7 Erlangen 91058 Germany; ^2^ College of Chemistry and Chemical Engineering Northeast Petroleum University Provincial Key Laboratory of Oil and Gas Chemical Technology Daqing 163318 China; ^3^ Department of Science and High Technology University of Insubria Via Valleggio 11 22100 Como Italy; ^4^ Department of Chemistry Faculty of Science King Abdulaziz University P.O. Box 80203 Jeddah 21569 Saudi Arabia

**Keywords:** dewetting and dealloying, H_2_ generation, photocatalysis, porous Pt, TiO_2_ nanotubes

## Abstract

Pt nanoparticles are typically decorated as co‐catalyst on semiconductors to enhance the photocatalytic performance. Due to the low abundance and high cost of Pt, reaching a high activity with minimized co‐catalyst loadings is a key challenge in the field. We explore a dewetting‐dealloying strategy to fabricate on TiO_2_ nanotubes nanoporous Pt nanoparticles, aiming at improving the co‐catalyst mass activity for H_2_ generation. For this, we sputter first Pt‐Ni bi‐layers of controllable thickness (nm range) on highly ordered TiO_2_ nanotube arrays, and then induce dewetting‐alloying of the Pt‐Ni bi‐layers by a suitable annealing step in a reducing atmosphere: the thermal treatment causes the Pt and Ni films to agglomerate and at the same time mix with each other, forming on the TiO_2_ nanotube surface metal islands of a mixed PtNi composition. In a subsequent step we perform chemical dealloying of Ni that is selectively etched out from the bimetallic dewetted islands, leaving behind nanoporous Pt decorations. Under optimized conditions, the nanoporous Pt‐decorated TiO_2_ structures show a>6 times higher photocatalytic H_2_ generation activity compared to structures modified with a comparable loading of dewetted, non‐porous Pt. We ascribe this beneficial effect to the nanoporous nature of the dealloyed Pt co‐catalyst, which provides an increased surface‐to‐volume ratio and thus a more efficient electron transfer and a higher density of active sites at the co‐catalyst surface for H_2_ evolution.

## Graphical abstract

**Combined dewetting‐alloying and dealloying of metal thin films** provides reliable nanostructuring tools to form on TiO_2_ nanotubes a nanoporous Pt co‐catalyst that exhibits a significantly enhanced mass activity for photocatalytic H_2_ evolution.

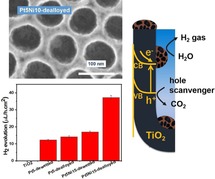

## Introduction

Since the pioneering work of Fujishima and Honda in 1972,^1^ the splitting of water by using light has received great attention as a potential mean for converting solar energy into clean and renewable vectors or fuels such as hydrogen gas.^2^, ^3^, ^4^, ^5^ In the field of photocatalysis and photo‐electrochemistry, TiO_2_ has attracted enormous research interest^6^, ^7^ due to a set of key advantages: TiO_2_ is in fact cheap, abundant, corrosion‐resistant, environmentally friendly and has a suitable conduction band edge to evolve H_2_ by reduction of H_2_O or other suitable agents.^8^, ^9^ However, the high recombination rate of photo‐generated electron‐hole pairs in the semiconductor as well as the low rate of charge transfer to reactants at the solid–liquid junction typically result in a low photon‐to‐product conversion efficiency. This, along with the relatively large band gap, impedes the development of cost‐effective TiO_2_ based photocatalytic and photo‐electrochemical units.

Many efforts have been therefore given in the last 40 years to modify TiO_2_ in view of enhancing its photo‐activity. An efficient strategy is to “decorate” the semiconductor surface with co‐catalyst nanoparticles of a suitable metal, such as Pt, Au or Pd, which can act as electron trapping sites to limit the electron‐hole pair recombination in TiO_2_. This can extend the lifetime of charge carriers and enhance the photocatalytic activity.^10^, ^11^, ^12^, ^13^, ^14^ Conduction band (CB) electron trapping occurs because high work function metals can form a rectifying Schottky barrier at the TiO_2_ surface. Moreover, metals such as Pt can also serve as catalytic cathodic sites for hydrogen atom recombination and H_2_ evolution.^15^ However, because of the high cost and low abundance of Pt, the understanding of the co‐catalyst morphology‐activity relationship can be key to design more efficient photocatalysts, providing for example a higher activity per mass of loaded co‐catalyst.

To this end, nanoporous metals have attracted considerable interest: their bicontinuous structure, tunable pore sizes, good electrical conductivity and high structural stability can lead to an improved performance in various applications, for example, in electrocatalysis, energy storage, or sensing.^16^, ^17^, ^18^, ^19^, ^20^, ^21^ In this context, dealloying is a most straightforward approach to produce nanoporous metals or porous metal NPs. Dealloying is based on the selective dissolution of a less noble metal component from a (single phase) metal alloy by chemical or electrochemical methods.^22^, ^23^ The dissolution of the less noble component is coupled with diffusion and aggregation of the more noble metal element at the solid/liquid interface: the overall process leaves behind a porous metal structure (a metal “sponge”) that can be enriched or mainly composed of the nobler element.^24^ Nanoporous metals prepared via selective dealloying of solid solutions possess a three‐dimensional (3D) structure of randomly interpenetrating ligaments/pores with sizes between a few nm to several tens of μm; these structural features can be precisely tuned by varying the preparation conditions (such as alloy composition, dealloying time, temperature, and electrochemical parameters) or by subsequent a thermal coarsening step.^18^, ^25^, ^26^, ^27^, ^28^, ^29^

In spite of the large application in heterogeneous catalysis, there is however still a comparably low number of studies on nanoporous metal co‐catalysts for photocatalytic applications. Nguyen et al. have reported on porous Au,^30^ AuPt,^31^ or PtPd^32^ nanoparticles produced on TiO_2_ nanotubes (NTs) by chemical dealloying of dewetted‐alloyed nanoparticles. These porous nanoparticles combined to TiO_2_ were found to strongly enhance the photocatalytic activity.

In this work, we fabricate a photocatalytic platform based on anodic TiO_2_ NTs functionalized with a porous Pt co‐catalyst formed via dewetting‐alloying‐dealloying^33^, ^34^, ^35^ of sputtered Pt‐Ni bilayers. For this we firstly sequentially coat the TiO_2_ NTs with a thin Pt and Ni metal film (few nm thick) by Ar‐plasma sputtering. Then we expose the Pt−Ni coated structures to a suitable thermal treatment to induce dewetting‐alloying of the Pt‐Ni bi‐layer.^36^ Due to metal atom surface mobility, the Pt and Ni films break up and mix with each other forming Pt−Ni dewetted‐alloyed islands. Afterwards these structures are exposed to a chemical dealloying treatment, under free‐corrosion conditions, to selectively remove Ni from the dewetted‐alloyed Pt‐Ni islands, thereby leaving behind a dealloyed porous Pt co‐catalyst at the TiO_2_ NTs surface.

The role of the Pt and Ni film initial thickness, the deposition sequence and dealloying time are herein investigated to achieve control over the final composition and morphology of the dealloyed Pt co‐catalyst. By optimizing these parameters, we produced nanoporous Pt/TiO_2_ structures that deliver a significantly enhanced photocatalytic H_2_ evolution performance compared to structures functionalized with comparable loadings of dewetted, non‐porous Pt. The improved photocatalytic performance is ascribed to the high specific surface area and nanoporous structure of the Pt co‐catalyst.

## Results and Discussion

**Scheme 1:**
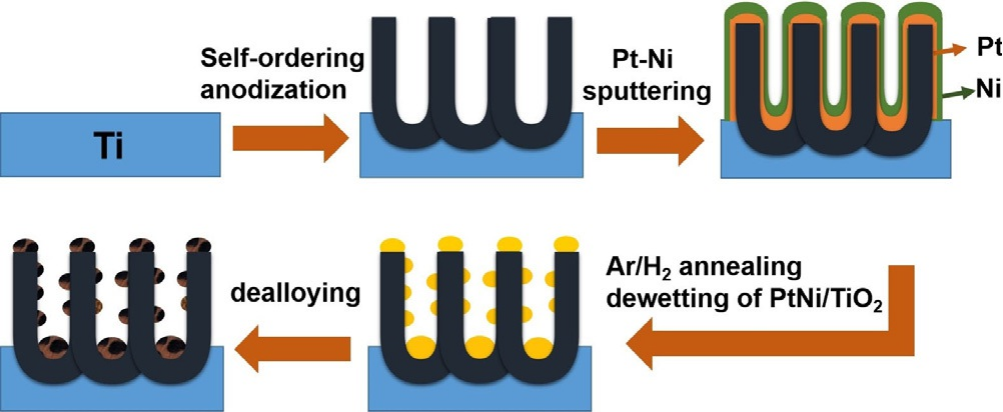
Fabrication of the dewetted‐dealloyed nanoporous Pt co‐catalyst at the surface of highly ordered TiO_2_ nanotube arrays.

The photocatalyst fabrication method adopted in this work is exemplified in Scheme 1; the experimental details can be found in the SI. As photocatalytic platform we use arrays of highly‐ordered TiO_2_ NTs. TiO_2_ NTs have attracted considerable interest in photocatalysis and photo‐electrochemistry in the last decades due to their reliable and versatile fabrication method, high surface area and one‐dimensional (1D) morphology;^37^, ^38^ namely the latter can provide attractive charge transport properties.^39^ Moreover, in the context of the present work, these highly‐ordered TiO_2_ nanotube layers can be used as a defined, corrugated photo‐active surface to host the formation of the nanoporous Pt co‐catalyst by a dewetting‐alloying‐dealloying approach.^34^, ^35^, ^40^

**Figure 1:**
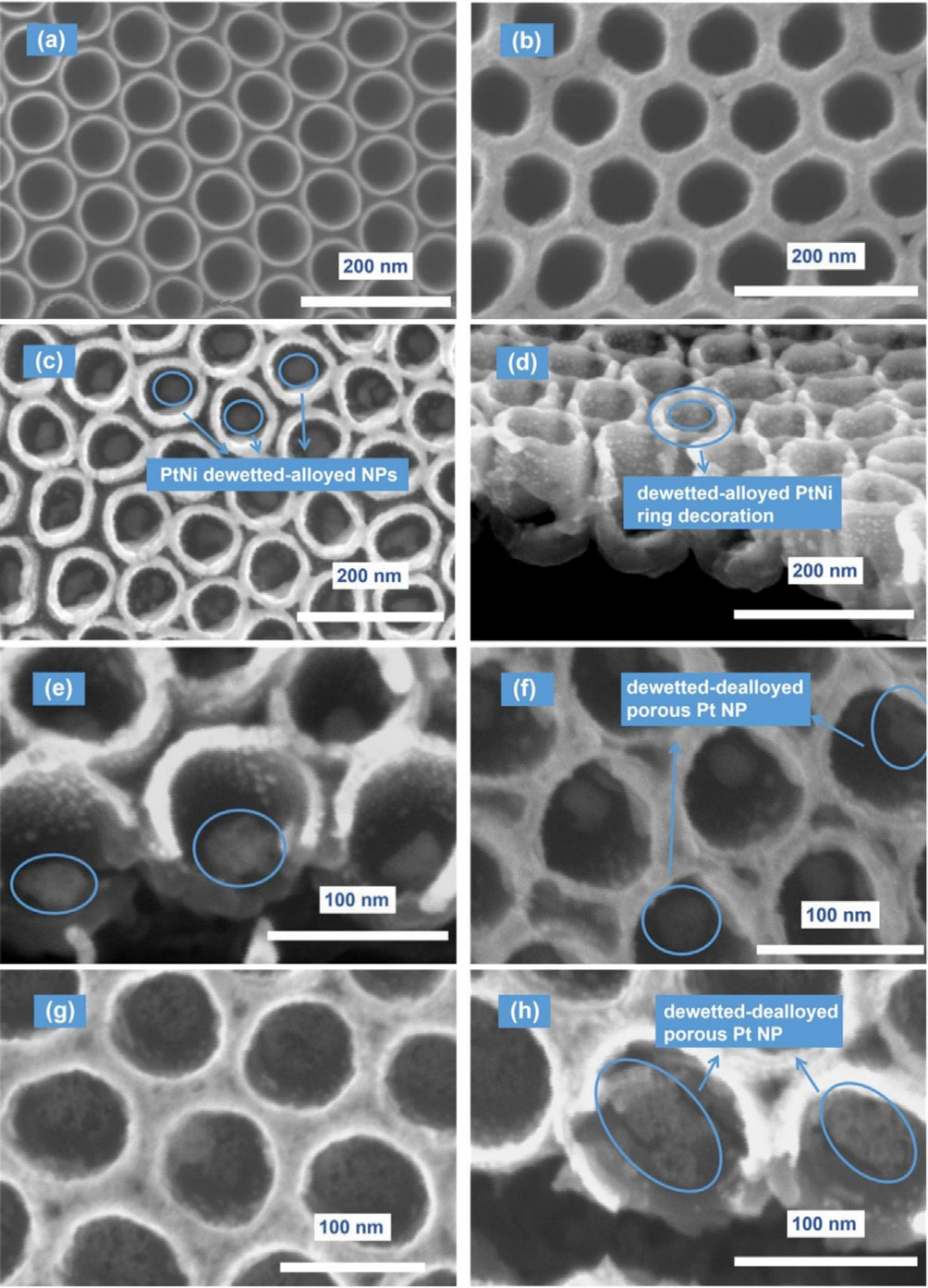
SEM images of: (a) highly ordered TiO_2_ NTs; (b) Pt5Ni10‐as‐sputtered; (c–d) Pt5Ni10‐dewetted; (e) Pt5Ni10‐dealloyed; (f) Pt5Ni15‐dealloyed; (g–h) Pt5Ni20‐dealloyed.

The TiO_2_ NTs were grown by self‐organizing electro‐chemical anodization of a Ti metal foil in a hot HF/*o*‐H_3_PO_4_ electrolyte.^41^ The SEM image in Figure 1 a shows the morphology of as‐prepared structures. These TiO_2_ NTs have an inner diameter of ≈70 nm and sidewalls with a thickness of ≈10 nm, while the NTs layer thickness (NT length) is ≈150 nm. The TiO_2_ NTs are then sputter‐coated (by Ar plasma sputtering) with thin Pt and Ni metal films. The metal coating is uniformly distributed over the nanotube surface, as one can see from Figure 1 b; here the structures are sequentially coated with a Pt film, nominally 5 nm thick, followed by a 10 nm thick Ni film.

We screened different Pt:Ni nominal ratios, by sputtering firstly the Pt film (5 nm) and then Ni (different thicknesses), this to assess the effect on the morphology of the dewetted state, the feasibility of dealloying the dewetted bimetallic islands, and the resulting photocatalytic H_2_ evolution activity. The samples are labelled as “PtxNiy”, where “x” and “y” indicate the nominal thickness of the sputtered Pt and Ni films, respectively. The adoption of this deposition sequence (namely Pt followed by Ni) is dictated by the fact that depositing Ni first seems to limit dewetting (metal film agglomeration) especially when using relatively thick Ni films (shown in Figure S1). This may be ascribed to the susceptibility of Ni to oxidize, for example, by interaction with the underneath oxide surface, resulting in a poor metal atom surface mobility.

To induce dewetting, that is, the rupture of the conformal metal film into metal islands or particles,^33^ the PtNi‐coated TiO_2_ structures were exposed to a thermal treatment in a 10 % H_2_/Ar atmosphere, at 450 °C for 1 h. For a bi‐layer of 5 nm Pt and 10 nm Ni, dewetting results in the formation at the TiO_2_ NT bottom of ≈40 nm sized PtNi NPs, while the dewetted metal bilayer forms at the NT mouths (NT top) a PtNi “continuous” decoration network (Figure 1 c–d). We chose a reducing atmosphere based on previous work where dewetting of metal films such as Ni, Co or Fe, which can be susceptible to oxidation, was carried out in H_2_ or in vacuum.^42^, ^43^, ^44^ The H_2_ partial pressure prevents oxidation of the metal film and provides the required atom surface mobility for metal agglomeration and for Pt‐Ni intermixing.

After dewetting, the PtNi‐decorated TiO_2_ NTs were immersed in an acidic mixture for 10 mins to induce dealloying, i.e the selective dissolution of Ni (see the Experimental Section for more details). Figure 1 e–h show the samples Pt5Ni10, Pt5Ni15 and Pt5Ni20 after dealloying. For samples Pt5Ni10 and Pt5Ni15 (Figure 1 e,f) and particularly for sample Pt5Ni20 (Figure 1 g,h) one can notice that the dewetted metal particles at the TiO_2_ NT bottom are converted through the dealloying step into nanoporous metal islands. We evaluated also the possibility of Pt losses during the dealloying step and concluded that only negligible amounts of Pt undergo dissolution—see the SI.

**Figure 2:**
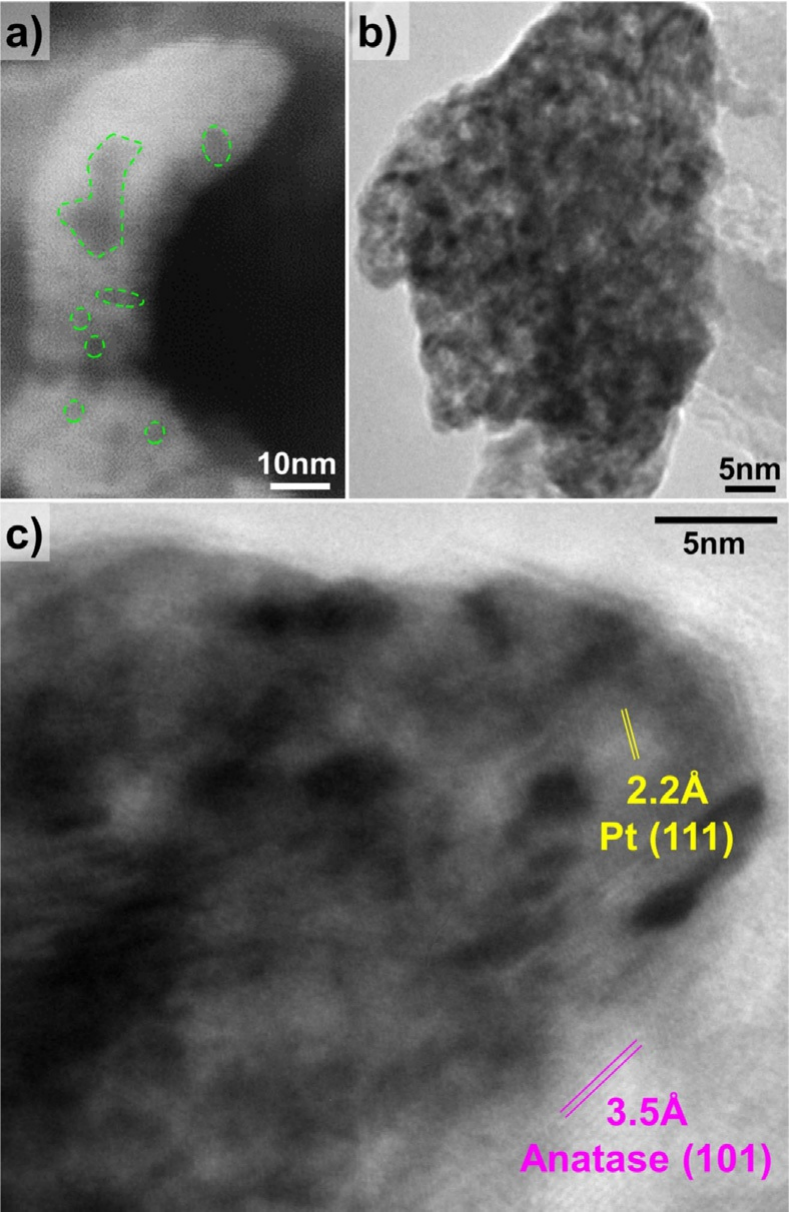
a) SEM and b,c) TEM characterization of the nanoporous Pt co‐catalyst for sample Pt5Ni10‐dealloyed.

To investigate the nanoporous nature of the dewetted‐dealloyed Pt co‐catalyst, we carried out TEM analysis for sample Pt5Ni15‐dewetted‐dealloyed, along with additional SEM investigation at high magnifications. The results are compiled in Figure 2. The high‐magnification SEM image in Figure 2 a shows that the dewetted‐dealloyed co‐catalyst is evidently porous, and the pores have a size is in the 3–20 nm range, with several very small pores (e.g. 3–5 nm) and few larger ones (>10 nm)—the pores are defined by dashed green lines. The TEM image in Figure 2 b confirms these results: the homogeneously distributed nm‐sized bright areas correspond to the pores in the Pt co‐catalyst particle. The higher resolution of TEM allows to detect also pores with a size of 1–2 nm. Moreover, the HR‐TEM image in Figure 2 c shows the lattice planes of Pt (111) and anatase TiO_2_ (101), with a spacing of 2.2 and 3.5 Å, respectively, which fit well to data in the literature.

**Figure 3:**
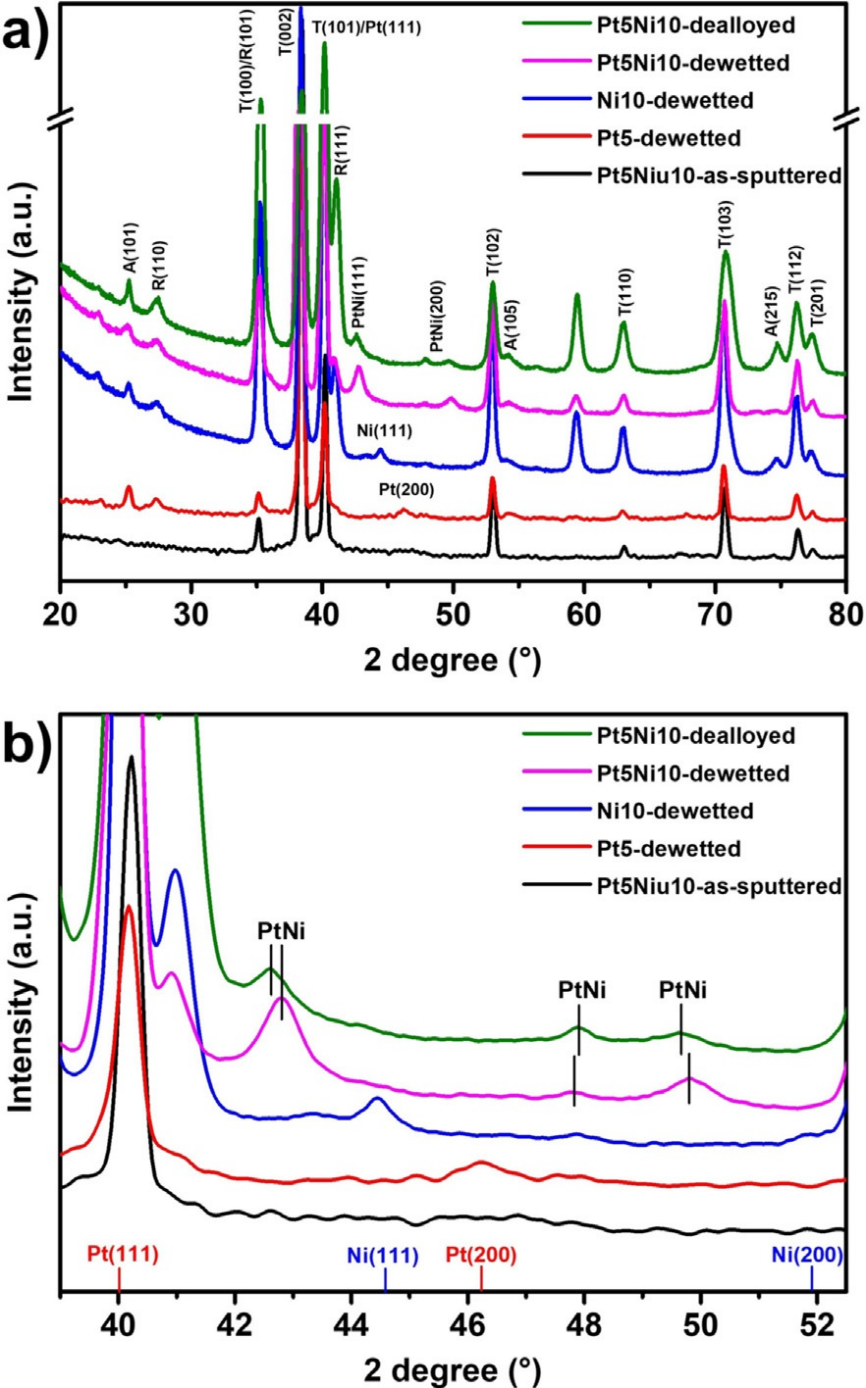
a) XRD patterns of Pt5Ni10‐as‐sputtered; Ni10‐dewetted; Pt5‐dewetted; Pt5Ni10‐dewetted and Pt5Ni10‐dealloyed; b) magnified view of the same patterns in the 40–52° 2*θ* region.

We characterized these structures by XRD to assess the crystallographic changes occurring during dewetting and dealloying. Figure 3 shows the XRD patterns of structures at different fabrication stages.

The XRD pattern of sample Pt5Ni10 before dewetting features only the reflections of Ti metal (due to the presence of the Ti metal substrate underneath the TiO_2_ NT structure). This confirms the amorphous nature of as‐anodized TiO_2_ NTs. The absence of Pt and Ni reflections in the as‐sputtered samples may on the one hand be ascribed to their low amount at the NT surface (below detection limit), or can on the other hand suggest that as‐sputtered Pt and Ni films are amorphous (or features extremely small crystalline domains, which can thus not be detected by XRD).

The same sample after dewetting shows a clearly different XRD pattern: firstly, the thermal treatment induces the crystallization of the NTs into a mixed anatase‐rutile TiO_2_ phase, as suggested by the appearance of reflections at 25.2° and 27.4° attributable to the (101) anatase (PDF no: 00‐021‐1272) and (110) rutile (PDF no: 00‐021‐1276) phases, respectively. More importantly, a reflection at 42.8° appears (i.e. between the theoretical Pt(111) and Ni(111) peaks) that can be ascribed to the formation of a Pt‐Ni alloy (Figure 3 b). This confirms that dewetting of Pt‐Ni bilayers forms nano‐sized Pt‐Ni alloy decorations at the TiO_2_ NT surface.^36^ Beside the Pt‐Ni (111) reflection, also the peaks at 47.9° and 49.8° (i.e. between the peaks of Pt (200) and Ni (200)) can be attributed to a Pt−Ni alloy phase. It is reported that the lattice constant of Pt‐Ni alloys is typically lower than that of pure Pt (3.92 Å) and higher than that of pure Ni (3.52 Å).^45^, ^46^, ^47^, ^48^, ^49^, ^50^ Thus, the peaks of Pt−Ni alloy phase are expected to be slightly shifted towards higher angles with respect to those of pure Pt phase, as in the case of our results.

The XRD data of samples Pt5 and Ni10 after dewetting provide a further confirmation that Pt‐Ni alloying occurs with dewetting. Here no peak attributable to Pt‐Ni alloy phases appears but for sample Ni10‐dewetted only an intense diffraction peak at 44.4° can be seen that fits well to the (111) plane of metallic Ni (JCPDS no.03‐065‐0380). This in general also supports that dewetting induces the agglomeration of the Ni film and crystallization/grain growth in the dewetted Ni islands. For sample Pt5‐dewetted, the main Pt reflection (i.e. (111)) cannot be seen owing to overlap with the strong (101) Ti peak. However, the formation of crystalline Pt domains upon dewetting is supported by the appearance of the Pt (200) reflection at 46.2°.

When comparing the XRD pattern of sample Pt5Ni10‐dealloyed with Pt5Ni10‐dewetted, it is possible to appreciate the chemical changes induced by the selective removal of Ni on the crystallographic feature of the formed nanoporous metal: firstly, the reflection associated to the Pt‐Ni alloy decreases in intensity, which can be ascribed to a partial amorphization of the dealloyed metal (this is in line with the rearrangement and diffusion of Pt atoms caused by Ni removal); secondly, such a peak slightly shifts from 42.8° to 42.6°, which confirms the removal of Ni and the consequent expansion of the metal lattice.^51^

**Figure 4:**
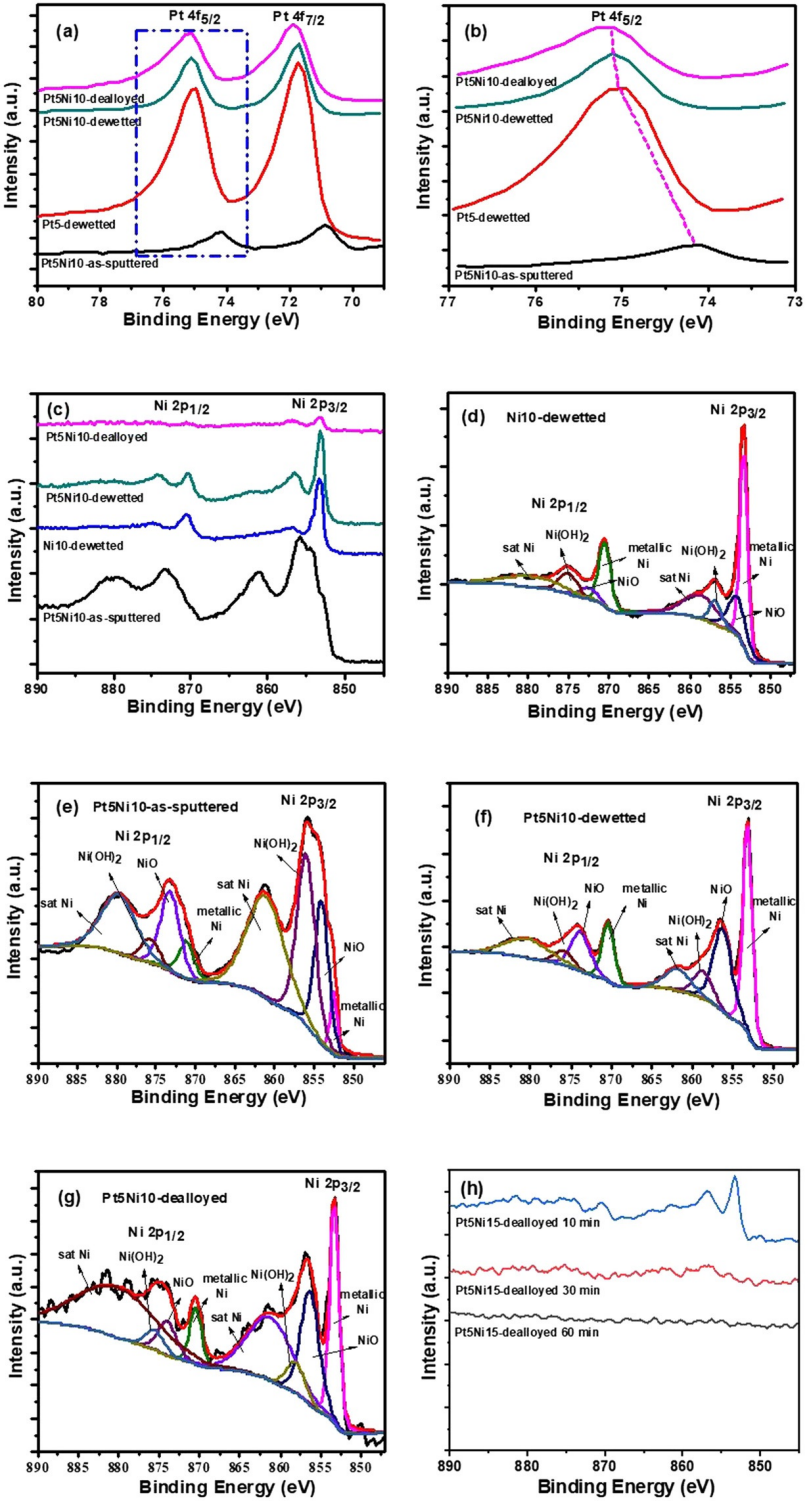
(a–c) Pt 4f and Ni 2p XPS high‐resolution spectra of Pt5Ni10‐as‐sputtered, Pt5‐dewetted, Pt5Ni10‐dewetted and Pt5Ni10‐dealloyed (etching time: 10 minutes); (d‐g) fitted XPS spectra in the Ni 2p region for samples Ni10‐dewetted, Pt5Ni10‐as‐sputtered, Pt5Ni10‐dewetted, and Pt5Ni10‐dealloyed (h) Ni 2p XPS high‐resolution patterns of Pt5Ni10‐dealloyed for different times.

We further characterized the structures by X‐ray photoelectron spectroscopy (XPS); as discussed below, the XPS data (Figure 4) complement the XRD results providing additional evidences of the occurrence of dewetting‐alloying and dealloying.

Figure 4 a shows the XPS spectra in the Pt 4f region for sample Pt5Ni10 at different fabrication stages, that is, as‐sputtered, after dewetting and after dealloying. We included the spectrum of sample Pt5 after dewetting. Compared to sample Pt5‐dewetted (reference for pure dewetted Pt), the spectrum of sample Pt5Ni10 as‐sputtered is substantially different: the low XPS signal intensity is caused by the fact that in this sample the Pt film is “buried”, that is, coated by the Ni film, while the significant peak shift towards lower B.E. values can be attributed to a strong interaction with both the underneath oxide and Ni overlayer.

On the contrary, both spectra of samples Pt5Ni10‐dewetted and Pt5Ni10‐dealloyed only slightly differ from the reference (Pt5‐dewetted). The spectrum of Pt5Ni10‐dewetted (thermal treatment in H_2_/Ar) shows the typical signature of Pt metal:^41^ that is, two sharp peaks at ≈75.1 and 71.7 eV associated to the Pt 4f_5/2_ and Pt 4f_7/2_ signals, respectively.^52^ This confirms the metallic state of Pt (Pt^0^) in particles dewetted from both Pt films or Pt‐Ni bilayers. Moreover, the Pt 4f peaks of Pt5Ni10‐dealloyed show a minor shift towards higher B.E. values (Figure 4 a) that is caused by the selective removal of Ni and is in agreement with results reported in previous literature.^53^

The XPS spectra in the Ni 2p region are compiled in Figure 4 c–g. Figure 4 c shows the spectra of sample Pt5Ni10 as‐sputtered, after dewetting and after dealloying (included is also the spectrum of sample Ni10 after dewetting, as a reference for dewetting of a pure Ni film). The fitting of the Ni 2p_3/2_ peak for sample Pt5Ni10 as‐sputtered (Figure 4 e) reveals a dominant contribution of NiO and Ni(OH)_2_, while the content of Ni metal is relatively low. The signals of metallic Ni, NiO and Ni(OH)_2_ peak at 852.5, 854.2 and 853.2 eV, respectively. These results can be ascribed to the high susceptibility of Ni to atmospheric oxygen, that is, the sputtered Ni metal films undergo immediate oxidation after sputtering when exposed to ambient conditions. However, the fitting of the spectra for sample Ni10 and Pt5Ni10 after dewetting (Figure 4 d,f) shows that the main contribution to the Ni 2p_3/2_ signal peaks at 853.2 eV, that is, the thermal treatment in H_2_/Ar causes the reduction of NiO and Ni(OH)_2_ to Ni metal. These results confirm the metallic state of Ni in the dewetted Pt‐Ni particles. The Ni 2p spectrum of sample Pt5Ni10‐dealloyed shows an evident reduction of the Ni signal (Figure 4 g), proving the selective removal of Ni from the dewetted bimetallic particles. This is also clear from the XPS surface elemental concentration compiled in Table 1 for sample Pt5Ni10 as‐sputtered, dewetted and dealloyed. Moreover, the spectra in Figure 4 h show that the longer the duration of the chemical dealloying, the lower the final Ni content, until reaching a complete removal of Ni after a 60 min long dealloying step.

**Table 1:** Surface elemental composition (atomic concentration, at.%) determined by XPS for the structures at different fabrication stages.

Sample	C 1s	N 1s	F1s	O 1s	Ti 2p	Ni 2p	Pt 4f
Pt5Ni10‐as‐sputtered	20.7	1.2	1.3	41.7	0.3	33.5	1.3
Pt5Ni10‐dewetted	50.9	0.0	0.5	28.1	1.3	13.5	5.8
Pt5Ni10‐dealloyed (10 min)	56.2	2.5	0.0	29.4	3.3	1.3	7.2

**Figure 5:**
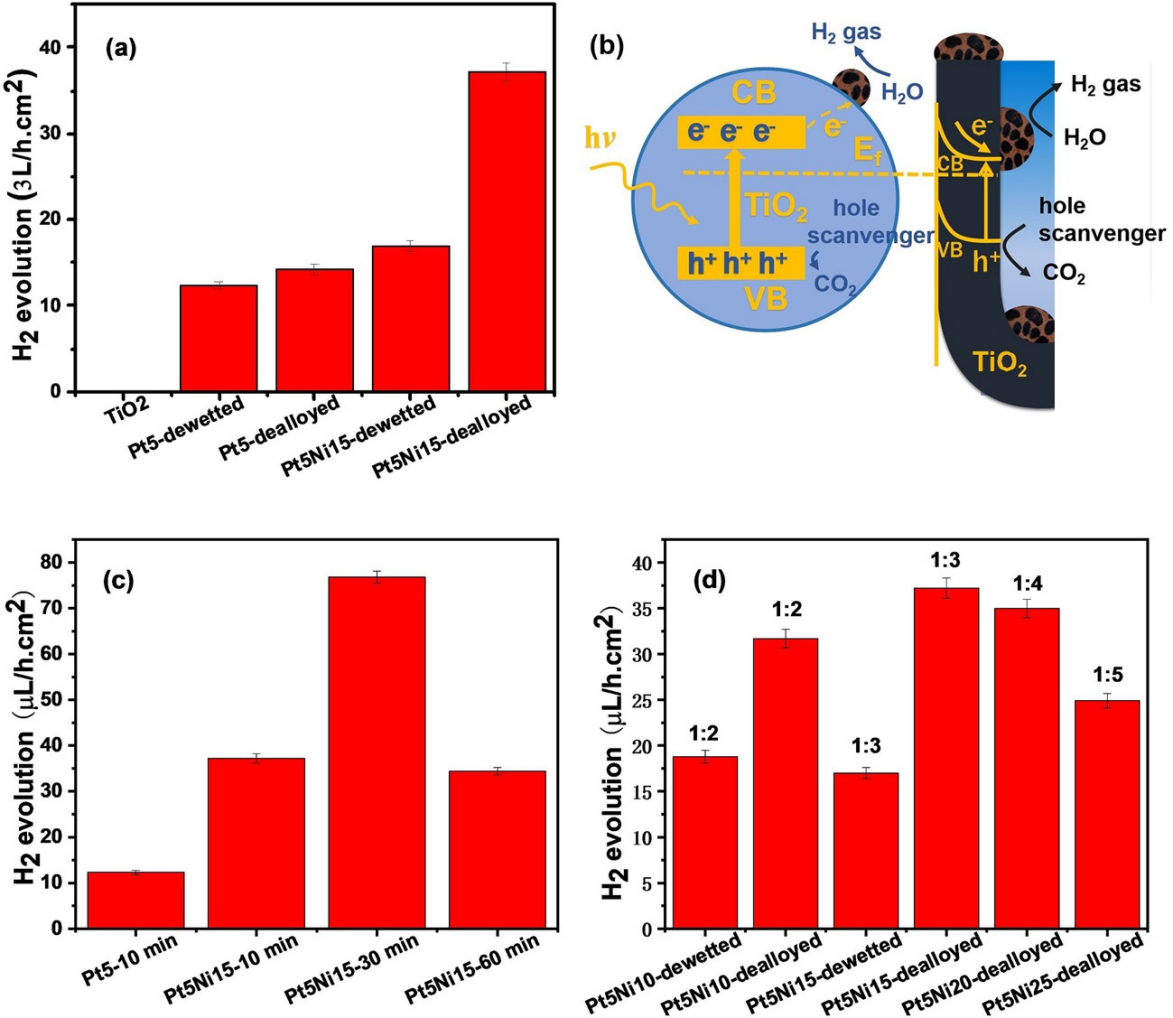
(a) Photocatalytic H_2_ evolution rate measured for pristine TiO_2_ NTs and TiO_2_ NTs decorated with dewetted or dewetted‐dealloyed (porous) Pt NPs; (b) Sketch of the photocatalyst structure; Photocatalytic H_2_ evolution rate measured for (c) Pt5Ni15‐dealloyed for different times at room temperature, and (d) TiO_2_ NTs decorated with dewetted or dewetted‐dealloyed (porous) Pt NPs form from bi‐layers of different composition and nominal thickness (i.e. different Pt:Ni ratio; dealloying time 10 minutes).

We finally investigated the photocatalytic H_2_ generation rate (r_H2_) of TiO_2_ NT structures decorated with different co‐catalysts, that is, dewetted Pt, dewetted Pt‐Ni and porous Pt; the data are compiled in Figure 5 a. The H_2_ evolution was measured in a gas tight quartz reactor, under UV light illumination (LED 365 nm, 100 mW cm^−2^) and in 20 vol. % ethanol aqueous solution.

The activity of pristine TiO_2_ NTs is negligible, that is, we measured a H_2_ evolution rate of 0.06 μL h^−1^ cm^−2^ in the absence of co‐catalyst. The NT layers used in the present work are rather thin, that is, ca. 200 nm‐thick, and thus although intense UV light is used for photo‐activation, only a poor photo‐activity is generally observed.^30^, ^41^, ^55^, ^56^ This can be due to the following factors i) the short thickness of the NT layer is not optimized to fully absorbed the incident photon flux,^56^, ^57^ and ii) a high photon flux in the absence of charge transfer co‐catalyst (Pt for electron transfer) may generate in the NTs a high density of photo‐generated charge carriers with high recombination rate (short‐lived carriers).

The decoration with dewetted Pt NPs leads to higher H_2_ evolution rates (12.2 μL h^−1^ cm^−2^), as also found in previous work.^58^, ^59^ This can be ascribed to the formation of a Pt/TiO_2_ Schottky junction that aids TiO_2_ CB electron extraction and transfer to the environment, as well as to the intrinsic catalytic activity of Pt for hydrogen recombination. The activity of NTs decorated with dewetted, pure Ni nanoparticles was investigated in a recent report of ours.^60^ Depending on the Ni film nominal thickness (e.g. 5–10 nm), the resulting r_H2_ was found to vary in the 0.2–0.5 μL h^−1^ cm^−2^ range; this confirms that the dewetted Ni co‐catalyst is significantly less active than pure, dewetted Pt. Interestingly, sample Pt5Ni15‐dewetted, that is, NTs functionalized with a Pt‐Ni alloy co‐catalyst, shows compared to dewetted Pt a slight improvement of the H_2_ evolution rate (17.1 μL h^−1^ cm^−2^), which can be ascribed to the bimetallic nature of the co‐catalyst as reported in previous work.^13^, ^61^, ^62^, ^63^

More importantly, the NTs decorated with porous Pt (Pt5Ni15‐10 minutes long dealloying) show a dramatic enhancement of the H_2_ evolution activity, reaching a production rate of 37.2 μL h^−1^ cm^−2^, which is nearly 4 times higher than that measured for the dewetted, pure Pt co‐catalyst (Pt5‐dewetted) in spite of the comparable nominal Pt loading.

Please note that control experiments proved that if an identical etching treatment (dealloying) is applied to sample Pt5‐dewetted, the resulting activity remains virtually unaltered (see the r_H2_ of sample Pt5‐dealloyed, Figure 5 a). Therefore the photocatalytic enhancement has to be ascribed to the nanoporous structure of the dealloyed Pt co‐catalyst, which provides an increased surface‐to‐volume ratio and thus a higher density of active site at the co‐catalyst surface for H_2_ evolution (Figure 5 b).^64^

We then explored the effect of the dealloying time on the photocatalytic performance. Remarkably, the data in Figure 5 c show that an increase of the dealloying time from 10 to 30 min further enhances the H_2_ evolution activity by a factor 2, hence reaching a production rate of 76.7 μL h^−1^ cm^−2^. An extended etching (60 min) however turned out to be detrimental, leading to a r_H2_ comparable to that of structures dealloyed for 10 min.

We have quantified the Pt loading based on ICP‐AES data reported in our previous work.^65^ A nominally 1 nm‐thick Pt film corresponds to a Pt loading of 1.41 μg cm^−2^. A (nominally) 5 nm‐thick Pt film (as in the case of the best performing sample Pt5Ni15‐dealloyed) corresponds to a loading of 7.05 μg cm^−2^. Thus, the H_2_ evolution activity per unit mass of loaded Pt is 10.9 and 1.7 μL_H2_  h^−1^ μg_Pt_^−1^ for samples Pt5Ni15‐dewetted‐dealloyed and Pt5‐dewetted, respectively. I.e. dealloyed (nanoporous) Pt leads to a 6.4 times higher activity compared to the non‐porous co‐catalyst, in spite of the same precious metal loading.

To confirm the beneficial effect of Pt porousification on the H_2_ evolution activity, we tested a series of photocatalysts produced by dewetting‐dealloying Pt−Ni bilayers of different nominal thicknesses and Pt:Ni ratios; the data are summarized in Figure 5 d. The results not only suggest that a Pt:Ni nominal thickness ratio of ≈1:3 is a most optimized condition to reach a high photocatalytic performance but also confirm that for the explored Pt‐Ni composition ratios the activity of the dealloyed, nanoporous co‐catalyst is superior than that of non‐porous co‐catalysts, that is, dewetted‐alloyed Pt‐Ni or dewetted, pure Pt decorations.

## Conclusions

We reported on the fabrication of TiO_2_ nanotube arrays functionalized with a nanoporous Pt co‐catalyst produced by a dealloying approach from dewetted‐alloyed Pt−Ni bilayers. For these structures we observed a superior photocatalytic H_2_ generation ability under UV light irradiation compared to nanotubes loaded with comparable amounts of non‐porous co‐catalysts such as pure, dewetted Pt or dewetted‐alloyed Pt−Ni decorations. The photocatalytic enhancement originates from the porous morphology of the Pt co‐catalyst, which can enable a more efficient electron transfer kinetics due to a large surface area and high density of surface active sites for H_2_ evolution. With the present work we provided insights for the synthesis of noble metal‐based (co‐)catalytic materials with high mass activity for solar energy conversion processes. In a broader context, the outlined concepts can in principle be extended to the fabrication of a large variety of nanoporous metals for application in catalysis, sensing or plasmonics.

## Experimental Section

### Fabrication of the TiO_2_ nanotube layers

Self‐organized highly ordered TiO_2_ nanotube arrays were in the present work formed on Ti substrates by means of electrochemical anodization. Briefly, prior to anodization, the titanium foils (1.5×2.5 cm^2^, Advent Research Materials, 0.125 mm thickness and 99.6 % purity) were cleaned by sonication in acetone, ethanol and deionized water for 15 minutes each step, respectively, followed by drying under a N_2_ gas stream. Then, the Ti foils were anodized to fabricate the highly ordered TiO_2_ nanotube arrays in a hot electrolyte based on 3 m HF in *o*‐H_3_PO_4_ (Sigma–Aldrich).^41^ For the anodization process, a two‐electrode configuration was used where the Ti foil and a Pt sheet were the working and counter electrodes, respectively. The anodization experiments were carried out by applying a potential of 15 V (for 2 h) using a DC power supply (VLP 2403 Voltcraft). The electrolyte was kept at 120 °C during anodization. After anodization, the samples were immersed in ethanol for 1 h to remove electrolyte remnants and were then dried under a N_2_ gas stream.

### Formation of the nanoporous Pt co‐catalyst

To form the nanoporous Pt co‐catalyst on TiO_2_ NTs, we used a sputter‐dewetting‐dealloying approach as follows:

⋅: Metal sputtering: a plasma‐sputtering machine (EM SCD 500, Leica) was used to sputter Pt and Ni metal thin films using a 99.99 % pure Pt target (Hauner Metallische Werkstoffe) and a 99.98 % pure Ni target (Hauner Metallische Werkstoffe). The applied sputtering current was 16 mA and the pressure inside the sputtering chamber was set to 10^−2^ mbar with Ar gas. As discussed below, the optimized sputtering sequence is Pt first followed by Ni. The amount of sputtered metals was in situ controlled by monitoring the metal film nominal thickness using an automated quartz crystal microbalance.
⋅: Thermal dewetting: the samples were annealed at 450 °C in a 10 % H_2_/Ar atmosphere (flux=3 L h^−1^) for 1 h, to induce dewetting of the Pt‐Ni bi‐layers and form alloyed PtNi decorations on the TiO_2_ NTs.
⋅: Dealloying: Dealloying was carried out in an acidic medium composed of 37.5 % HNO_3_ (70 %), 37.5 % acetic acid and 25 % H_2_SO_4_ (98 %), at room temperature for different dealloying times.

### Characterization of the structures

Field‐emission scanning electron microscope (FESEM Hitachi S4800) and high resolution transmission electron microscope (HRTEM, Philips CM30) were employed to characterize the morphology of the samples. X‐ray diffraction (XRD) performed with a X′pert Philips MPD (equipped with a Panalytical X′celerator detector) using graphite monochromized Cu_Kα_ radiation (*λ*=1.54056 Å) was used to analyze the crystallographic properties of the samples. The chemical composition of the samples was examined by X‐ray photoelectron spectroscopy (XPS, PHI 5600 US) and peak positions were calibrated with respect to the C 1s peak at 284.8 eV.

### Photocatalytic measurements

For the photocatalytic runs we followed a protocol described in previous work.^66^ The H_2_ generation experiments were carried out under illumination with UV light (UV‐LED smart Opsytec, 365 nm, 100 mW cm^−2^) for 6 h. The samples were immersed in an aqueous solution of ethanol (20 vol. %) in a quartz tube cell that was sealed with a rubber septum. Before running the photocatalytic experiments, the quartz cell was purged with N_2_ gas to remove O_2_. 200 μL gas samples were withdrawn at the end of the photocataytic experiments and analyzed by gas chromatography (GCMS‐QO2010SE, Shimadzu) to examine the amount of evolved H_2_. The GC was equipped with a thermal conductivity detector (TCD), a Restek micropacked Shin Carbon ST column (2 m×0.53 mm). GC measurements were conducted at a temperature of 45 °C (isothermal conditions) with the temperature of the injector setup at 280 °C and that of the TCD at 260 °C. The flow rate of the carrier gas (Argon) was 14.3 mL min^−1^.

## Conflict of interest

The authors declare no conflict of interest.

## Supporting information

As a service to our authors and readers, this journal provides supporting information supplied by the authors. Such materials are peer reviewed and may be re‐organized for online delivery, but are not copy‐edited or typeset. Technical support issues arising from supporting information (other than missing files) should be addressed to the authors.

Supplementary
