# Supplementary material for: A Dewetted‐Dealloyed Nanoporous Pt Co‐Catalyst Formed on TiO2 Nanotube Arrays Leads to Strongly Enhanced Photocatalytic H2 Production
Source: Chem Asian J. 2020 Jan 7;15(2):301–9. doi: 10.1002/asia.201901545 (PMC7004064; doi:10.1002/asia.201901545)
Supplement: Supplementary file 1 — Supplementary [file ASIA-15-301-s001.pdf]

CHEMISTRY
AN ASIAN JOURNAL

Supporting Information

A Dewetted-Dealloyed Nanoporous Pt Co-Catalyst Formed on TiO_2 Nanotube Arrays Leads to Strongly Enhanced Photocatalytic H_2 Production

Lei Ji,[a, b] Davide Spanu,[a, c] Nikita Denisov,[a] Sandro Recchia,[c] Patrik Schmuki,*[a, d] and Marco Altomare*[a]

asia_201901545_sm_miscellaneous_information.pdf

Figure S1 SEM images of samples Pt3Nix-dewetted and NixPt3-dewetted.

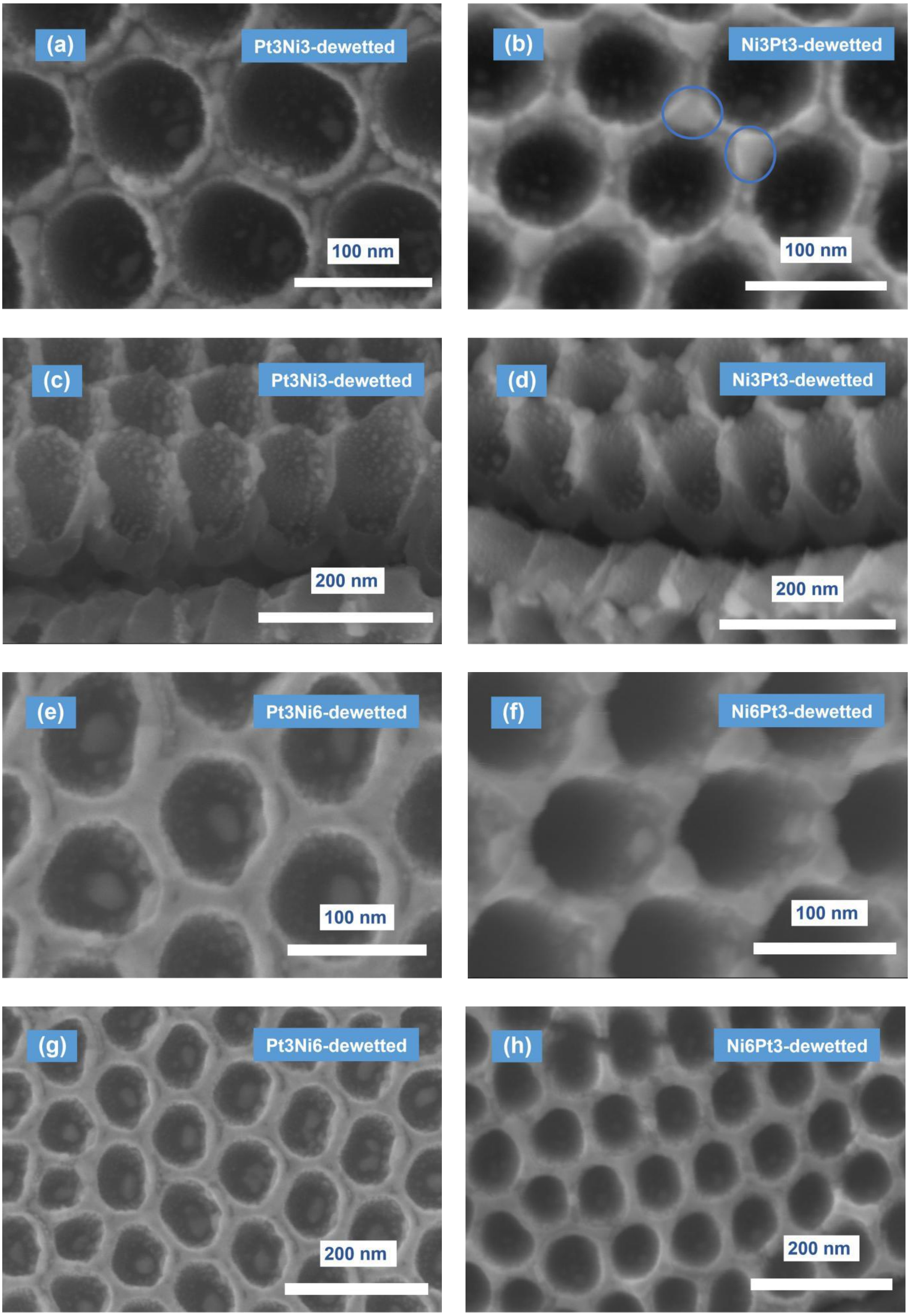

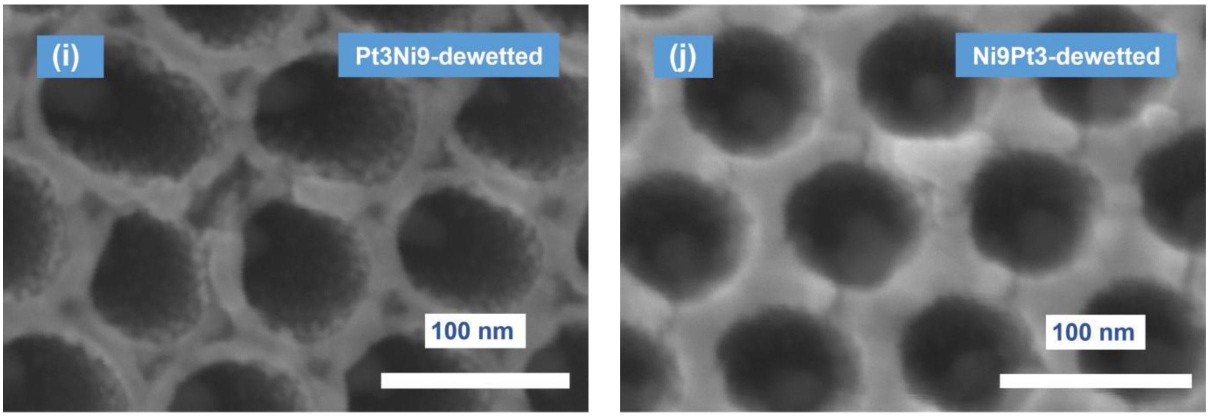

To evaluate possible Pt losses during dealloying, we proceeded as following:

We prepared a fresh dewetted-alloyed sample (Pt5Ni15-dewetted-alloyed), which we than dealloyed following our standard procedure. The dealloying solution was transferred into the photocatalytic cell (quartz tube); we immersed in this solution a pristine TiO_2 NT sample (previously crystallized at 450°C, in air for 1h, to make it photo-active) and then purged the solution and the cell head-space with Ar gas for 30 minutes (to remove O_2). Afterwards we illuminated the TiO_2 NT sample for 30 min with UV light provided by a 365 nm LED (100 mW cm^{-2}), aiming at photo-depositing Pt ions that could be present in the solution due to (possible) Pt dissolution during the dealloying step.

After photo-deposition we characterized the sample by XPS and measured its photocatalytic H_2 evolution activity. XPS data (Fig. S2) show a weak signal in the B.E. region between 80 and 68 eV (i.e. Pt 4f region); this signal can be attributed to photo-deposited Pt. Nevertheless, the Pt surface content is in this case below the XPS detection limit (typically 0.03at%[1]), suggesting that only negligible amounts of Pt were lost (dissolved) during the dealloying process. Moreover, the H_2 evolution activity of this sample resulted comparable to that of pristine NT arrays (i.e. 0.06 μL h^{-1} cm^{-2}).

Figure S2 XPS analysis of TiO_2 NTs after photo-deposition in the etchant used for dealloying.

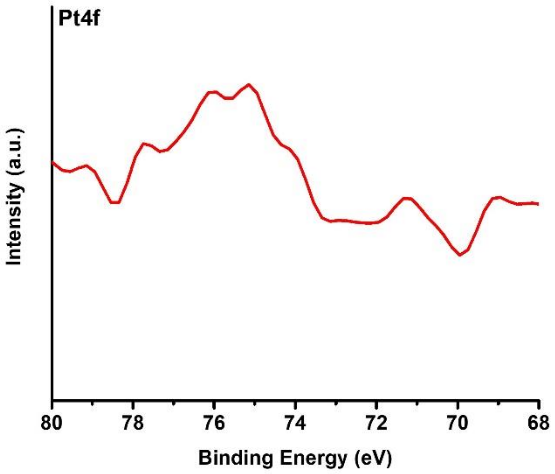

References

[1] A. G. Shard, *Surf. Interface Anal.* **2014**, *46*, 175.
